# Supplementary material for: Differential protection by nicotinamide in a mouse model of glaucoma DBA/2J revealed by second-harmonic generation microscopy
Source: PLoS One. 2024 Sep 10;19(9):e0309400. doi: 10.1371/journal.pone.0309400 (PMC11386466; doi:10.1371/journal.pone.0309400)
Supplement: S1 Table — (DOCX) [file pone.0309400.s001.docx]

**S1 Table.** **The measurement values of D2 and D2-*Gpnmb+*.**

| **#** | **Strain** | **NAM** | **Age** | **Gender** | **IOP1** | **IOP2** | **IOP3** | **SHG density** | **Volume** |
| --- | --- | --- | --- | --- | --- | --- | --- | --- | --- |
| 1 | D2 | - | 7.2 | F | 10 | 10 | 11 | 174.37 | 10.3 |
| 2 | D2 | - | 7.2 | F | 9 | 10 | 10 | 186.96 | 9.5 |
| 3 | D2 | - | 8.1 | F | 9 | 9 | 9 | 45.41 | 8.6 |
| 4 | D2 | - | 8.1 | F | 8 | 9 | 8 | 107.33 | 11.6 |
| 5 | D2 | - | 8.3 | F | 10 | 10 | 11 | 167.14 | 7.6 |
| 6 | D2 | - | 8.3 | F | 11 | 10 | 10 | 284.60 | 8.4 |
| 7 | D2 | - | 9.2 | F | 9 | 10 | 10 | 109.27 | 8.2 |
| 8 | D2 | - | 9.2 | F | 10 | 10 | 10 | 135.83 | 5.7 |
| 9 | D2 | - | 9.7 | F | 12 | 11 | 12 | 50.24 | 8.3 |
| 10 | D2 | - | 9.7 | F | 10 | 11 | 11 | 221.11 | 10.3 |
| 11 | D2 | - | 9.9 | F | 11 | 11 | 11 | 187.25 | 9.9 |
| 12 | D2 | - | 10.9 | F | 11 | 12 | 11.5 | 7.56 | 0.5 |
| 13 | D2 | - | 10.9 | F | 12 | 13 | 12 | 72.97 | 1.2 |
| 14 | D2 | - | 10.9 | F | 11 | 11 | 10 | 203.53 | 4.2 |
| 15 | D2 | - | 10.9 | F | 12 | 12 | 11 | 25.21 | 1.2 |
| 16 | D2 | - | 11.5 | F | 13 | 14 | 13 | 27.00 | 1.6 |
| 17 | D2 | - | 11.5 | F | 11 | 12 | 12 | 254.22 | 4.3 |
| 18 | D2 | - | 11.5 | F | 11 | 11 | 11 | 94.63 | 0.6 |
| 19 | D2 | - | 12.4 | F | 13 | 13 | 14 | 1.97 | 0.7 |
| 20 | D2 | - | 13.0 | F | 12 | 13 | 13 | 17.36 | 2.2 |
| 21 | D2 | - | 7.6 | M | 11 | 10 | 12 | 228.40 | 4.6 |
| 22 | D2 | - | 8.8 | M | 11 | 11 | 10 | 109.74 | 9.7 |
| 23 | D2 | - | 9.0 | M | 10 | 9 | 12 | 167.70 | 9.7 |
| 24 | D2 | - | 9.0 | M | 12 | 12 | 14 | 143.04 | 2.8 |
| 25 | D2 | - | 9.0 | M | 9 | 10 | 10 | 48.64 | 7.4 |
| 26 | D2 | - | 9.0 | M | 10 | 10 | 9 | 234.82 | 10.6 |
| 27 | D2 | - | 10.1 | M | 11 | 11 | 10 | 94.27 | 8.8 |
| 28 | D2 | - | 10.1 | M | 11 | 11 | 10 | 182.07 | 11.0 |
| 29 | D2 | - | 10.3 | M | 13 | 15 | 14 | 69.94 | 11.9 |
| 30 | D2 | - | 11.0 | M | 10 | 11 | 10 | 230.95 | 1.8 |
| 31 | D2 | - | 11.0 | M | 10 | 11 | 10 | 119.37 | 6.9 |
| 32 | D2 | - | 12.8 | M | 12 | 12 | 14 | 81.11 | 6.0 |
| 33 | D2 | - | 12.8 | M | 14 | 16 | 17 | 50.92 | 7.8 |
|  |  |  |  |  |  |  |  |  |  |
| **#** | **Strain** | **NAM** | **Age** | **Gender** | **IOP1** | **IOP2** | **IOP3** | **SHG density** | **Volume** |
| 1 | D2 | + | 7.1 | F | 10 | 10 | 10 | 153.73 | 6.9 |
| 2 | D2 | + | 7.1 | F | 12 | 11 | 12 | 286.76 | 7.7 |
| 3 | D2 | + | 7.9 | F | 10 | 9 | 11 | 189.31 | 11.1 |
| 4 | D2 | + | 9.0 | F | 9 | 9 | 8 | 92.89 | 8.0 |
| 5 | D2 | + | 9.0 | F | 10 | 10 | 9 | 174.71 | 8.3 |
| 6 | D2 | + | 9.2 | F | 10 | 11 | 9 | 190.46 | 8.6 |
| 7 | D2 | + | 9.4 | F | 11 | 11 | 12 | 109.14 | 8.8 |
| 8 | D2 | + | 9.4 | F | 10 | 10 | 11 | 164.88 | 10.5 |
| 9 | D2 | + | 9.7 | F | 10 | 11 | 10 | 116.38 | 3.9 |
| 10 | D2 | + | 9.7 | F | 10 | 10 | 11 | 214.41 | 8.5 |
| 11 | D2 | + | 9.9 | F | 10 | 10 | 11 | 224.11 | 11.1 |
| 12 | D2 | + | 9.9 | F | 11 | 11 | 12 | 115.64 | 3.8 |
| 13 | D2 | + | 10.2 | F | 10 | 11 | 11 | 207.37 | 8.8 |
| 14 | D2 | + | 10.2 | F | 11 | 10 | 11 | 152.16 | 12.1 |
| 15 | D2 | + | 10.4 | F | 11 | 12 | 12 | 129.93 | 4.4 |
| 16 | D2 | + | 10.4 | F | 10 | 11 | 11 | 159.58 | 10.7 |
| 17 | D2 | + | 10.9 | F | 9 | 10 | 10 | 22.13 | 0.3 |
| 18 | D2 | + | 10.9 | F | 10 | 11 | 11 | 10.26 | 0.7 |
| 19 | D2 | + | 11.3 | F | 12 | 12 | 11 | 185.80 | 8.3 |
| 20 | D2 | + | 11.3 | F | 11 | 11 | 12 | 185.11 | 6.5 |
| 21 | D2 | + | 11.5 | F | 12 | 10 | 12 | 9.61 | 1.5 |
| 22 | D2 | + | 11.5 | F | 11 | 11 | 12 | 18.05 | 1.6 |
| 23 | D2 | + | 11.8 | F | 13 | 8 | 10 | 93.31 | 9.5 |
| 24 | D2 | + | 11.8 | F | 12 | 9 | 13 | 42.66 | 3.0 |
| 25 | D2 | + | 12.3 | F | 12 | 11 | 14 | 21.09 | 10.2 |
| 26 | D2 | + | 12.3 | F | 11 | 10 | 10 | 9.01 | 0.8 |
| 27 | D2 | + | 12.8 | F | 12 | 12 | 12 | 97.64 | 13.4 |
| 28 | D2 | + | 12.8 | F | 11 | 12 | 13 | 182.11 | 10.2 |
| 29 | D2 | + | 13.1 | F | 12 | 12 | 10 | 150.36 | 7.2 |
| 30 | D2 | + | 13.1 | F | 12 | 10 | 10 | 209.30 | 8.3 |
| 31 | D2 | + | 8.3 | M | 8 | 9 | 9 | 102.24 | 9.0 |
| 32 | D2 | + | 8.3 | M | 9 | 10 | 9 | 153.56 | 7.6 |
| 33 | D2 | + | 8.5 | M | 8 | 8 | 9 | 124.17 | 12.3 |
| 34 | D2 | + | 8.5 | M | 9 | 9 | 8 | 150.11 | 11.3 |
| 35 | D2 | + | 8.8 | M | 10 | 9 | 10 | 96.93 | 11.9 |
| 36 | D2 | + | 10.6 | M | 9 | 9 | 8 | 168.37 | 8.8 |
| 37 | D2 | + | 10.6 | M | 11 | 10 | 10 | 167.17 | 6.0 |
| 38 | D2 | + | 11.3 | M | 11 | 10 | 11 | 91.86 | 6.1 |
| 39 | D2 | + | 12.4 | M | 12 | 12 | 13 | 48.27 | 2.8 |
| 40 | D2 | + | 12.6 | M | 11 | 9 | 10 | 163.33 | 10.0 |
| 41 | D2 | + | 13.0 | M | 12 | 13 | 12 | 105.79 | 10.8 |
|  |  |  |  |  |  |  |  |  |  |
|  |  |  |  |  |  |  |  |  |  |
| **#** | **Strain** | **NAM** | **Age** | **Gender** | **IOP1** | **IOP2** | **IOP3** | **SHG density** | **Volume** |
| 1 | D2-*Gpnmb+* | - | 6.2 | F | 9 | 8 | 8 | 172.74 | 7.3 |
| 2 | D2-*Gpnmb+* | - | 6.2 | F | 10 | 9 | 9 | 153.68 | 9.0 |
| 3 | D2-*Gpnmb+* | - | 8.8 | F | 9 | 8 | 9 | 134.36 | 15.9 |
| 4 | D2-*Gpnmb+* | - | 8.8 | F | 10 | 9 | 11 | 144.18 | 12.7 |
| 5 | D2-*Gpnmb+* | - | 9.2 | F | 8 | 9 | 10 | 193.49 | 10.5 |
| 6 | D2-*Gpnmb+* | - | 12.5 | F | 10 | 10 | 11 | 275.03 | 7.9 |
| 7 | D2-*Gpnmb+* | - | 5.5 | M | 9 | 10 | 11 | 183.04 | 12.7 |
| 8 | D2-*Gpnmb+* | - | 5.5 | M | 10 | 11 | 12 | 124.42 | 10.6 |
| 9 | D2-*Gpnmb+* | - | 7.6 | M | 10 | 9 | 9 | 199.48 | 9.0 |
| 10 | D2-*Gpnmb+* | - | 7.6 | M | 10 | 8 | 8 | 154.72 | 10.1 |
| 11 | D2-*Gpnmb+* | - | 10.2 | M | 8 | 8 | 8 | 309.19 | 10.5 |
| 12 | D2-*Gpnmb+* | - | 10.2 | M | 9 | 8 | 9 | 316.87 | 10.0 |
| 13 | D2-*Gpnmb+* | - | 11.0 | M | 8 | 9 | 9 | 326.72 | 4.0 |
| 14 | D2-*Gpnmb+* | - | 12.3 | M | 10 | 12 | 11 | 123.22 | 7.2 |
| 15 | D2-*Gpnmb+* | - | 14.6 | M | 10 | 10 | 11 | 207.82 | 8.3 |
| 16 | D2-*Gpnmb+* | - | 14.6 | M | 9 | 9 | 9 | 200.82 | 6.9 |
| 17 | D2-*Gpnmb+* | - | 15.9 | M | 8 | 9 | 9 | 243.27 | 6.1 |
| 18 | D2-*Gpnmb+* | - | 15.9 | M | 9 | 9 | 10 | 266.72 | 5.8 |
| 19 | D2-*Gpnmb+* | - | 16.1 | M | 10 | 9 | 10 | 270.57 | 7.0 |
