# Supplementary figures and images for: Differential protection by nicotinamide in a mouse model of glaucoma DBA/2J revealed by second-harmonic generation microscopy
Source: PLoS One. 2024 Sep 10;19(9):e0309400. doi: 10.1371/journal.pone.0309400 (PMC11386466; doi:10.1371/journal.pone.0309400)

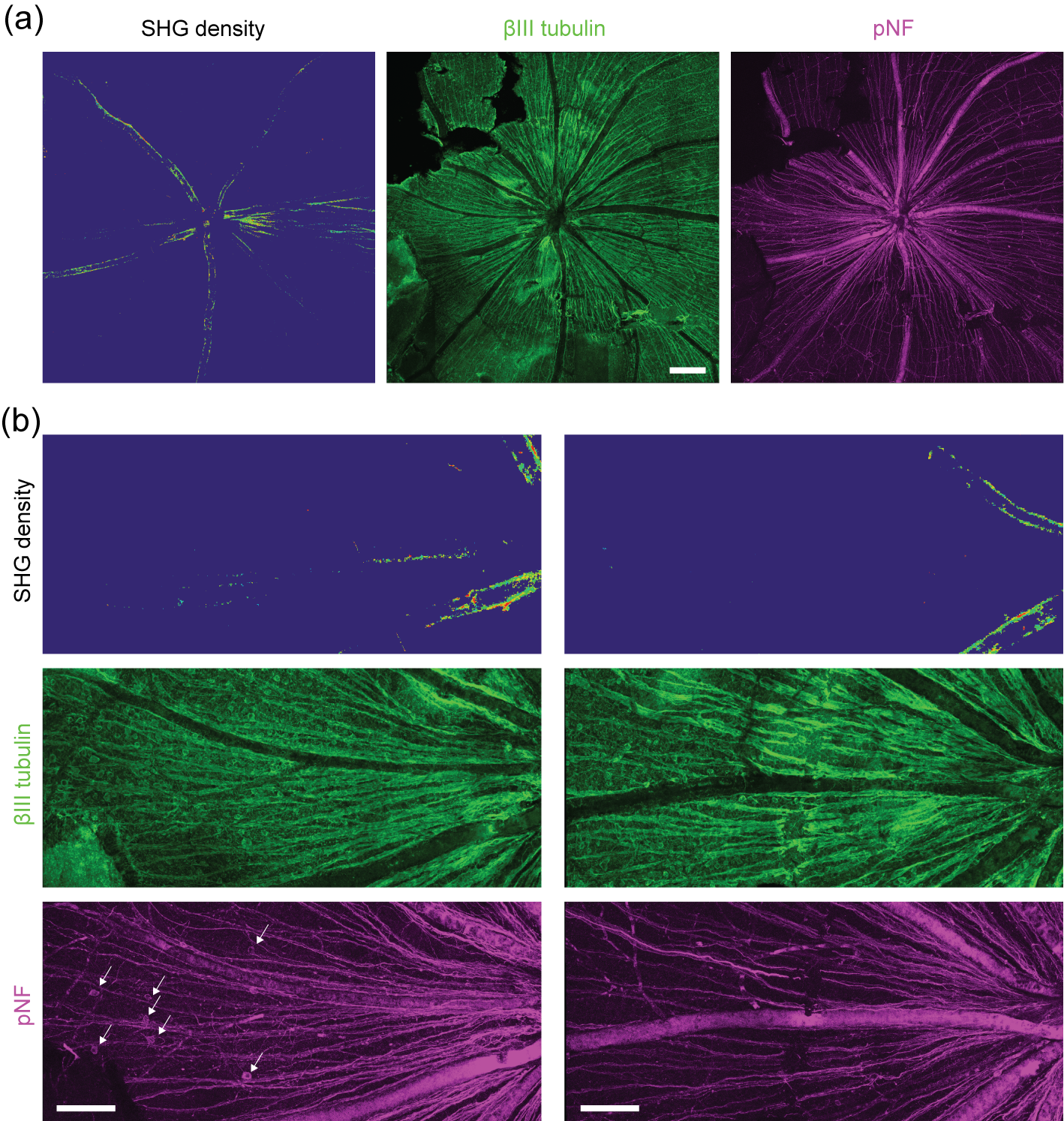

Supplement: S1 Fig — (a) Mosaics of a D2 retina (12 months age, female) by SHG and immunofluorescence against the cytoskeletal elements, βIII tubulin and pNF. Scale bars, 200 μm. (b) Comparing two sectors in different stages of RGC degeneration. Arrows, pNF+ RGC somas. Scale bars, 100 μm. (TIF) [file pone.0309400.s002.tif]
