# Supplementary material for: Differential protection by nicotinamide in a mouse model of glaucoma DBA/2J revealed by second-harmonic generation microscopy
Source: PLoS One. 2024 Sep 10;19(9):e0309400. doi: 10.1371/journal.pone.0309400 (PMC11386466; doi:10.1371/journal.pone.0309400)
Supplement: Attachment — Submitted filename: Response to Reviewers_Boodram and Lim.docx [file pone.0309400.s003.docx]

We are grateful for the thoughtful, expertly comments provided by the editor and reviewers. They have allowed us to identify areas of weakness in the previous version where our findings weren’t described adequately for the general readership. We revised the manuscript extensively with more detailed technical information to improve the rigor. We believe all the raised concerns are addressed. We received the following common criticisms from both reviewers:

1. IOP measurements with TonoLab

Both reviewers raised concerns about the IOP measurements. The IOP values of D2 mice we measured with TonoLab were lower than those in the literature. We used TonoLab according to the manufacturer’s instructions. Since the machine does not allow the user any control over calibration, other than choosing mouse or rat in the settings, the reason for the difference is unclear to us. Nonetheless, we concluded the measurements were reliable because 1) the measured IOPs on three different dates were consistently very close and 2) they displayed anticipated overall trends, such as D2 mice with age and NAM diet, as well as vs. D2-*Gpnmb+*. Individual IOP readings, which reviewer #2 asked about, are included as a Supplementary information in the revised version (S1 Table). Given the high repeatability/reproducibility, it seems unlikely that the underestimation was due to the low n (either number of samples or measurements), as reviewer #1 suspects. Rather, it could be a result of incorrect software setting or mechanical deviations of the rebound tonometer (which might be also behind the wide variability found in the literature). Conceivably, it is possible to determine, for a particular instrument that we used, the relationship between the actual and measured IOPs by performing a linear regression with the ground truths (e.g., by manometer). However, while the presumed scaling error does not affect the overall conclusion of our study, new errors that are harder to correct might arise from the extra steps of calibration. Considering these factors, the IOP values are presented as originally obtained with our TonoLab device, but a new paragraph is added to the Discussion regarding the accuracy of IOP measurement, as reviewer #2 recommended.

2. N, the number of eyes

Both reviewers pointed out the ambiguity of what N means. All N in the manuscript indicates eye samples, which we clarified in the revision. Reviewer #1 asked why the number of eyes was less than twice the number of animals and whether it was due to some selection. It is not the case. Not all eye samples yielded data because of the stringent conditions of SHG imaging. SHG signal from axonal microtubules lasts only for a few hours after enucleation, then it gradually decreases (presumably as the cytoskeleton depolymerizes in the absence of active regulation). No fixation protocol is yet known to preserve the signal. Consequently, there is a limited time window within which the imaging experiment must be completed. Furthermore, the quality of fresh, unfixed flatmount preparation is crucial for acquiring stacks of microscopic images across the large region. For the rigor of data, we discarded the retina samples that either took too long to prepare the flatmount or the full 3D volume could not be captured. Because of these demanding requirements, the success rate of SHG imaging was less than 100%. This information is added to the Methods.

3. DBA/2J (D2): Nomenclature and Animal section

DBA/2J (or D2) are used to denote the strain, as both reviewers suggested. Reviewer #1 asked about the acclimation. DBA/2J mice were brought in from The Jackson Lab at the ages of 8-12 weeks and housed until the desired age. Breeding pairs of DBA/2J-*Gpnmb+* were obtained at the age of around 4 weeks and the offsprings were raised to the desired age. The information is now included in the Methods.

4. Figure legends

Both reviewers commented that figure captions are insufficient. We made substantial improvements in the revision.

Other comments:

5. (Reviewer #1) D2 variability and experimental error

Our description of the experimental errors was misunderstood, i.e., “Overall, the variations of D2 data were greater than those of *Gpnmb+* representing primarily the experimental errors.”. The sentence is rewritten to clarify this.

6. (Reviewer #1) Microtubule deficit vs. PERG and anterograde axonal transport of CT-β

Reviewer #1 noted that other labs have shown that NAM prevents loss of axon transport and recovers PERG (PMID: 32605122, 28209901). Assuming that PERG is more correlated with the integrity of RGC axons (i.e., morphology) than microtubule cytoskeleton, it is understandable that NAM diet improves PERG signal. We can also reconcile the recovered anterograde axonal transport with our finding, i.e., that the mean SHG density, which is proportional to the density of microtubules per axon caliber, is not significantly protected by NAM as follows: Between NAM vs. no-NAM D2 retinas, as compared in the previous studies (Williams *et al.*), there are more total microtubules in the former, despite similarly low densities of microtubules, just because there are more RGC axons. Provided that the spatial extent of CT-β signals (both in the ONH and LGN) is correlated with the total amount of microtubules rather than with the density, it is expected that the NAM D2 retina will display more widespread CT-β signals than the no-NAM D2 retina. Based on our data, however, individual RGCs of the NAM D2 retina still suffer microtubule deficit. So, the CT-β signals are predicted to be much less intense in individual cells of the NAM D2 retina than those of the non-glaucomatous control (which Williams *et al.* did not compare). Furthermore, our results show that microtubule deficit (i.e., low mean SHG density) continues to worsen with age, even with NAM diet. Consequently, we predict that axonal transport in the NAM D2 retina will be compromised eventually at an age when the microtubule density drops below a certain level, regardless of the integrity of RGC axons. We included this as a paragraph in the Discussion.

7. (Reviewer #1) Background for the hypothesis of the study

Reviewer #1 asked for an appropriate context leading to the present study. The question, in our opinion, is related to the editor’s comment about our insufficient explanation of "morphology" vs "microtubules". We addressed this by including Fig. 1(c), as suggested, and clarifying the concepts in the revised text.

8. (Reviewer #1) Staging of D2 glaucoma

Reviewer #1 asked about the staging of D2 glaucoma. Unfortunately, our experimental design did not include the measurement of RGC counts. Only IOP and the properties of RGC axons, i.e., volume and mean SHG density, were measured as continuous independent variables, and their relationships were examined with age. The volume of the retinal nerve fibers can be considered an optical surrogate of RGC axon counts. We discuss this as one of the limitations of our study in the revision.

9. (Reviewer #1) NAM administration

Reviewer #1 questioned about NAM administration. NAM was added to the standard Rodent LabDiet 5001, the same diet the D2 mice received before switching to NAM-containing diet. For a dosage of 550 mg nicotinamide/kg body weight per day, we included 2750 mg nicotinamide/kg diet based on an average 25-g mouse consuming 5 g of diet per day. This information is added to the Methods.

10. (Reviewer #2) Last few sections of Results are not related to NAM treatment.

Reviewer #2 noted that the last two sections are not related to NAM treatment, which, considering that the title is “Protective effects of nicotinamide…”, should be modified. We agree with this critique. Those sections seek to investigate the details of microtubule deficit, or decoupling of morphology and microtubules, which was also evident in the response to NAM treatment. To address this comment, we changed the title to “Differential protection by nicotinamide…” and the sections are revised accordingly.

11. (Reviewer #2) Limited discussion

There were two related comments by Reviewer #2 regarding the limited discussion, i.e., that figure 6 is too simplistic and that the sentence “NAM promotes the morphology of the retinal nerve fibers more significantly than microtubules.” should be clarified. In response, we revised Fig 6 and the caption, and rewrote the first paragraph of Discussion entirely to enhance clarity.

Reviewer #2 encouraged us to include “a more thoughtful interpretation of the data particularly in relation to previous studies in multiple models – beyond the DBA model.”. In response, we added a new discussion comparing our finding of persistent microtubule deficit with the previously reported recovery of axonal transport by NAM. We also included a paragraph in the Discussion on how other glaucoma models beyond DBA/2 model could be beneficial for mechanistic studies of the NAM neuroprotection.

12. (Reviewer #2) Lines 151-152: Overstated conclusion regarding the origin of microtubule deficit.

The overstated conclusion is corrected.
