# Supplementary material for: Differential protection by nicotinamide in a mouse model of glaucoma DBA/2J revealed by second-harmonic generation microscopy
Source: PLoS One. 2024 Sep 10;19(9):e0309400. doi: 10.1371/journal.pone.0309400 (PMC11386466; doi:10.1371/journal.pone.0309400)
Supplement: Attachment — Submitted filename: Response to Reviewers_Boodram and Lim.docx [file pone.0309400.s005.docx]

We thank the reviewers for their thoughtful comments. We made every effort to understand the concerns and revised the manuscript to address all of them as summarized below.

(Reviewer 1) *I suggest that you add text pointing out that Gpnmb+ mice on NAM diet were not included as a control group but could have been informative.*

The text is included in the Discussion, as suggested. In hindsight, the control group could have informed us whether the IOP-independent effect of NAM is specific to the glaucoma process or also present in the non-glaucomatous eyes.

(Reviewer 1) *Please add a post hoc power analysis for at least one of the phenotypes and discuss the power briefly in the Discussion.* *Or, if the analyses are still in a discovery phase in which meaningful power analyses might be premature, please expand on what was learned from the current experiment that might guide future quantitative studies and point out the uncertain power of the current experiments in the caveats.*

Admittedly, we were not aware of the concept of post hoc power analysis prior to this comment. We found a fair amount of literature and online discussions on the topic, especially whether it is a meaningful analysis. Focusing on our study, we understand the question in hand is this: Given that we cannot reject the assumption that NAM diet does not have an effect on the mean SHG density (i.e., the null hypothesis), is there a sample size that we can calculate with which the effect could have been observed? The underlying reasoning is that the effect was not detected probably because it was too small and will be detectable with a larger sample size (or statistical power). The answer to the question is, yes, there can be such a sample size and, no, it cannot be calculated based on the current data. The explanation why such an estimation is flawed/misleading can be found in numerous papers (e.g., PMID: 11310512, 31552383, 29994928). In a nutshell, the estimated post hoc power is based on the variance of sample whereas the prospective power is on the variance of population. Because the estimated effect size is noisy, the power necessary to detect it (i.e., the post hoc power) is not informative. It has been proposed to present confidence intervals as an alternative (PMID: 11310512). This information is already in Fig.2, where the 95% confidence intervals are depicted around the slope of age-dependence for the two cases of diet.

(Reviewer 2) *Please state that IOP by cannulation is the gold standard for the DBA/2J model and errors in tonolab can arise due to changes to the eyes with age.*

The statement is added to the Discussion, as suggested.

(Reviewer 2) *The discussion about the IOP-independent mechanisms should be fleshed out a little further, possibly simply with further reference to the Williams et al Science paper indicating protection at the level of mitochondrial abnormalities.*

In response, we included a description of molecular changes reversed by NAM treatment, as shown in Williams et al., and the implications in the IOP-independent mechanism.

(Reviewer 2) *I do not think Figure 6 is necessary and should be removed.*

Figure 6 is removed, as suggested.
